# A first-generation microsatellite linkage map of the ruff

**DOI:** 10.1002/ece3.830

**Published:** 2013-10-24

**Authors:** Lindsay L Farrell, Terry Burke, Jon Slate, David B Lank

**Affiliations:** 1 Department of Animal and Plant Sciences, University of SheffieldSheffield, S10 2TN, U.K; 2 Department of Biological Sciences, Simon Fraser UniversityBurnaby, British Columbia, Canada, V5A 1S6

**Keywords:** Chromosomes, genetic map, linkage groups, microsatellite, ruff

## Abstract

A linkage map of the ruff (*Philomachus pugnax*) genome was constructed based on segregation analysis of 58 microsatellite loci from 381 captive-bred individuals spanning fourteen breeding years and comprising 64 families. Twenty-eight of the markers were resolved into seven linkage groups and five single marker loci, homologous to known chicken (*Gallus gallus*) and zebra finch (*Taeniopygia guttata*) chromosomes. Linkage groups range from 10.1 to 488.7 cM in length and covered a total map distance of 641.6 cM, corresponding to an estimated 30–35% coverage of the ruff genome, with a mean spacing of 22.9 cM between loci. Through comparative mapping, we are able to assign linkage groups Ppu1, Ppu2, Ppu6, Ppu7, Ppu10, Ppu13, and PpuZ to chromosomes and identify several intrachromosomal rearrangements between the homologs of chicken, zebra finch, and ruff microsatellite loci. This is the first linkage map created in the ruff and is a major step toward providing genomic resources for this enigmatic species. It will provide an essential framework for mapping of phenotypically and behaviorally important loci in the ruff.

## Introduction

Uniquely among birds, ruffs (*Philomachus pugnax*) exhibit three different and distinct permanent alternative male reproductive morphs, with correlated differences in territorial lekking behavior, body size, and the presence or coloration of ornamental breeding plumage. All populations include: (1) dark-plumed territorial “Independents,” (2) white-plumed nonterritorial “Satellites,” and (3) small female mimics called “Faeders” (Hogan-Warburg 1966; Höglund and Lundberg 1989; Van Rhijn 1973; Jukema and Piersma 2006). Status as an independent or satellite has been previously shown to be due to a genetic polymorphism in male mating behavior consistent with a single-locus, two-allele autosomal Mendelian mode of inheritance (Lank et al. 1995). More recently, it has been discovered that a dominant autosomal allele controls development in to female-mimicking faeders (Lank et al. 2013).

With the current evidence for Mendelian genetic determination of behavioural type (Lank et al. 1995) and a strong genetic basis also suspected for plumage characters (Dale et al. 2001), the ruff presents an ideal species for the study of functional genetic variation underlying phenotypic traits. However, genomic resources for the ruff are limited; only nine previously published microsatellite markers were available (Thuman et al. 2002) until the recent publications of Farrell et al. (2012) and Verkuil et al. (2012). As a step toward developing genomic resources for the ruff and to allow mapping of phenotypic traits, we performed linkage analysis of 58 microsatellites from 381 captive individuals comprising 64 families, and present here the resulting linkage map.

## Methods

### Mapping population

The genetic mapping population consisted of 381 individuals belonging to a captive population maintained by DBL over fourteen breeding years at Simon Fraser University, Canada. This population was established from 31 individuals raised from eggs collected on breeding grounds near Oulu, Finland in 1985, to which 63 additional wild birds were added during the years up to 1990. In 2006, two faeders, one satellite male, and one female captured in the Netherlands were added to the captive population. The pedigree used in this project contains individuals from 64 families, with 62 fathers and 93 mothers, with hatch years extending from 1985 for the original parental generation to 2009 for the most recent chicks. Breeding records held by DBL and genotyping of several loci by SB McRae (SBM; East Carolina University) determined parentage prior to this study.

### Microsatellite markers

In total, 102 microsatellite markers were tested, of which 52 were found to be polymorphic and were developed and characterized (Farrell et al. 2012). Forty-seven of these were selected for linkage mapping and used together with 11 ruff loci previously developed for population genetic studies (Thuman et al. 2002; Verkuil et al. 2012), and 5 other shorebird loci identified from cross-utility testing in the ruff and many other avian species (Saether et al. 2007; St. John et al. 2007; Küpper et al. 2008; Blomqvist et al. 2010; Dawson et al. 2010), which had all been tested previously in the current population (Lank et al. 2013; S. B. McRae, unpublished). There is as yet no reference genome for the ruff; therefore, to verify the position of each microsatellite marker and ensure adequate spacing and complete genome coverage, we predicted microsatellite locations for all markers in both the chicken and zebra finch genome assemblies (Table 1) by performing a search for sequence similarity using BLAST software via the ENSEMBL interface (http://www.ensembl.org), following approaches described elsewhere (Dawson et al. 2006, 2007). Chromosomal positions were plotted and visualized using MAPCHART (Voorrips 2002). Sequence data relating to the 63 markers were input into MULTIPLEX MANAGERv.1.0 (Holleley and Geerts 2009) to optimize marker reactions and create 13 multiplex panel sets that were then used to genotype the 381 individuals contained within the ruff pedigree (Table 1).

**Table 1:** Summary of genotyping results (58 loci) and predicted genome locations (53 loci) of ruff microsatellite markers

Locus	Locus reference	Fluoro-label	PCR *MP* set	CH chr ZF chr	Chicken locus Zebra finch locus^1^	*E*-value in Chicken *E*-value in Zebra finch	Repeat motif	*n*	*A*	*T*_a_ (°C)	Primer Sequence 5′–3′	Allele size range (bp)	*H*_O_	*H*_E_	Est. null allele freq.
Ppu001	Farrell et al. (2012)	HEX	7	1 1A	52975585 50776302	1.90E−138 3.30E−27	(TAGA)_12_	227	7	56	F: ACCAGGCTTCTTCCCTCTGGA R: TGAAACTTCACATTTTGGGGATGA	266–291	0.59	0.64	0.0519
Ppu003	Farrell et al. (2012)	HEX	9	1 1	122413141 13670975	5.60E−61 1.20E−27	(CTAT)_11_	296	6	56	F: CAGGATTGCTTTGGCTGGAG R: AGCATGTAGTGCTTCAGTTATTTAGATGC	365–374	0.59	0.56	−0.0268
Ppu005	Farrell et al. (2012)	6-FAM	4	8 8	22771586 19350034	8.00E−108 4.40E−73	(TC)_5_	287	8	56	F: GGAGCAATGTGATACCACTAAGGACTG R: CTCCTGACCTCCACCGCAAC	217–233	0.39	0.57	0.2050*
Ppu006	Farrell et al. (2012)	6-FAM	1	5 5	17130526 16041469	2.70E−53 8.10E−70	(GT)_9_	370	3	58	F: TGGAAGTGGAAGGAGGTCTGTG R: TCCACTCAGGTGCAGGCTTC	245–254	0.46	0.49	0.0319
Ppu007	Farrell et al. (2012)	6-FAM	9	3 3	76776326 76352543	4.00E−27 4.50E−52	(TG)_5_	295	4	56	F: GCCAGAGTAGCAACAGTCAGTGC R: CCTATTCATGTCTCCAAGTTCAATCC	281–294	0.51	0.53	0.0171
Ppu008	Farrell et al. (2012)	6-FAM	7	– –	– –	–	(CACA)_6_	227	4	56	F: GAAGTTCCTCTTACCAATTTGCTTGC R: TGACCTGCTGGTACTCCACCAC	295–301	0.22	0.22	0.0076
Ppu009	Farrell et al. (2012)	HEX	7	4 4	23020195 29737180	5.90E−64 3.70E−46	(ACAC)_6_	173	16	56	F: TCTTTATGATGCTATTTGAGGGTTTGG R: AATGCCACTGCACCAGAAGTAGTC	419–472	0.73	0.88	0.0924
Ppu011	Farrell et al. (2012)	HEX	1	3 3	57218799 49883043	3.40E−63 6.40E−43	(CA)_5_	361	4	58	F: CGCACATCTGCTGTTGAGAAATC R: TGGACTGAAGGTGACTATTCTGCTG	215–224	0.48	0.45	−0.0423
Ppu013	Farrell et al. (2012)	HEX	8	3 3	18786830 18163845	1.40E−61 3.80E−63	(AG)_6_	304	2	57	F: ACATGCTCCTCTTCCATTTGCAG R: TGCTCCATGGAATCAAACATGG	222–229	0.53	0.49	−0.0435
Ppu014	Farrell et al. (2012)	6-FAM	7	– 24	– 3112345	– 1.20E−30	(GTGT)_5_	227	3	56	F: CAACCCCATCTCCTGGCTTTT R: CAGCTCGGTACATTGGTGCTTG	207–220	0.51	0.45	−0.0639
Ppu015	Farrell et al. (2012)	HEX	4	2 2	19888521 22065196	9.80E−27 1.70E−54	(CA)_5_	300	5	56	F: GGTCCAGTTCTGTGTGCCAGTTT R: TGACTTTGGAGGTTGTTACTTATTGTTGTC	242–247	0.48	0.64	0.1588
Ppu016	Farrell et al. (2012)	HEX	12	1 1	166535076 65490386	1.10E−22 5.70E−13	(TCTC)_6_	224	8	60	F: TCAGGCAGTGGGACTAGATGATTG R: TCAAAGACTTCTGCAAAGTTATTCTTCTAAGC	212–229	0.66	0.66	−0.0037
Ppu017	Farrell et al. (2012)	HEX	11	4 –	52883524 –	4.30E−11 –	(TT)_7_	179	3	61	F: GTTGGCCTGGACTCCGTCTG R: GTGCTACTGAAATCGTCTGATGTTGG	227–229	0.02	0.48	0.9122*
Ppu018	Farrell et al. (2012)	HEX	9	2 2	61061152 71904153	1.90E−28 4.90E−38	(AGAT)_13_	281	9	56	F: TGCCTTCTTACTTTCTCAATATTTGTGG R: AGAGATACAGTAAGCTTCGTATGACAGACAC	242–274	0.79	0.79	0.0014
Ppu019	Farrell et al. (2012)	HEX	2	3 –	84720681 –	7.60E−10 –	(CA)_11_	343	7	61	F: TAACCCACGAGTGGCTCTG R: GCTACTGGGTGCTGTTACTTCC	145–162	0.77	0.78	0.0128
Ppu020	Farrell et al. (2012)	HEX	11	11 11	19600964 71134	3.10E−20 6.70E−25	(GT)_13_	335	5	61	F: TCCTGTCCTGTCCTTGGAAC R: GCGGTATTTCTGGCCTAGC	241–249	0.50	0.48	−0.0126
Ppu021	Farrell et al. (2012)	6-FAM	6	1 1	156510069 61096046	3.90E−57 4.00E−81	(CTAT)_12_	207	7	62	F: AAAGCTTGTAAGCTCTAAGCAATACC R: AGGCTATTGACACTTCACAAAGG	284–329	0.72	0.75	0.0085
Ppu022	Farrell et al. (2012)	6-FAM	13	2 2	75106465 79772128	8.90E−20 1.20E−37	(ATAGAT)_9_	315	8	63	F: TGAATGCATGAATTAGGTAGTGG R: GGGAAACATCATGCAACAAC	264–302	0.86	0.85	−0.0055
Ppu024	Farrell et al. (2012)	HEX	6	13 –	9775634 –	1.80E−12 –	(TCTA)_7_	205	10	62	F: GGAAACCTTCCCATCAACAG R: GAAGGGATGCATGGTTGG	122–161	0.79	0.85	0.0307
Ppu025	Farrell et al. (2012)	6-FAM	13	1 –	24109016 –	2.20E−18 –	(CA)_17_	313	9	63	F: GATCCAGACTGCCTAAACAGC R: GCATCACAAATGCAACTTCAG	332–352	0.86	0.85	−0.0102
Ppu027	Farrell et al. (2012)	6-FAM	12	7 7	15230709 19299021	1.20E−28 6.60E−43	(AAGA)_8_	232	12	60	F: TGTTAGCAGGCTGATGTGTG R: TCCTGTGAGCTGTTAATTCTGAG	281–379	0.61	0.67	0.0550
Ppu028	Farrell et al. (2012)	HEX	2	1 1	130142524 108220670	2.50E−12 2.30E−14	(TGAT)_6_	366	4	61	F: CCTGAACCATTAGTTTACTTGCTG R: GCACCAGAACTGCCACATAG	185–197	0.65	0.62	−0.0181
Ppu029	Farrell et al. (2012)	HEX	11	10 10	9185554 7745116	1.50E−50 1.10E−62	(TG)_10_	251	5	61	F: AGGGTATTGTTGGAGAAATGG R: CTAACCTGGATGGCTGTTTG	164–170	0.07	0.21	0.4942*
Ppu030	Farrell et al. (2012)	HEX	11	2 2	120354672 122215464	2.30E−77 1.30E−110	(TG)_11_	312	6	61	F: CAGGCTTAACACTCTTTCTTCC R: CTCGTTGGTCATAATTTGAGG	130–140	0.53	0.57	0.0318
Ppu031	Farrell et al. (2012)	6-FAM	5	13 13	1071128 16024608	2.30E−25 8.90E−41	(GT)_10_	355	4	59	F: TGATTCTTATTAGGATTATTTGATGC R: TGAGGACTGTGGTTTAAGAGC	319–326	0.32	0.30	−0.0400
Ppu032	Farrell et al. (2012)	6-FAM	7	2 2	29908799 49636829	6.50E−10 1.70E−05	(CA)_18_	209	9	56	F: CATTTCTTGTTGTGATTAATAGTCTCC R: TAAGAGGTTGCCAGGTTGTG	248–266	0.66	0.83	0.1094
Ppu034	Farrell et al. (2012)	HEX	3	10 10	12101849 10268721	6.40E−37 3.20E−56	(AAT)_6_	374	3	61	F: CTCCATGGACCAGAAATGAG R: CCACCCTTCATATTGACTCG	126–135	0.15	0.16	0.0176
Ppu036	Farrell et al. (2012)	6-FAM	1	10 10	4306840 895537	2.10E−33 1.20E−46	(TG)_7_	365	3	58	F: AGACCCGGGTGTTCAAGGTG R: TTTCCCAGCATGACATACATTGC	200–209	0.47	0.48	0.0114
Ppu037	Farrell et al. (2012)	HEX	13	26 26	2193405 1368934	1.10E−19 3.30E−50	(TG)_6_	337	2	63	F: CTCTTGTGGTACCTGGAAGAGGTG R: TCCATATTTATTACAGCCCAGAAGACC	234–236	0.34	0.32	−0.0316
Ppu038	Farrell et al. (2012)	HEX	12	2 2	98252724 101346526	1.70E−42 4.10E−22	(GAAA)_5_	230	3	60	F: CATGACTACCTATCGAATCCTCTTTGG R: TTAATATGGCAGCCTTACCTAACGAAAC	274–282	0.20	0.19	−0.0171
Ppu039	Farrell et al. (2012)	6-FAM	13	1 1A	52147383 49840927	1.50E−73 1.30E−52	(TGAT)_6_	336	3	63	F: GCAACTGCTGCACTCCCAAC R: CTTGCCATTCAGGTTAAGTACACTTCC	186–194	0.52	0.51	−0.0147
Ppu040	Farrell et al. (2012)	HEX	9	5 5	2524315 321716	1.20E−59 1.90E−58	(TG)_9_	291	5	56	F: CTCCTGGCTGCGTTGTTCTG R: GGAACGATGTGGGTTACTTCCAG	203–213	0.38	0.36	−0.0205
Ppu041	Farrell et al. (2012)	HEX	7	11 11	10001495 15593478	6.50E−56 1.90E−71	(AC)_9_	226	3	56	F: TGATTTTCCGAAACAAGTTTTAATCG R: AGCAGACCGGAGAAGCAACA	171–174	0.28	0.31	0.0499
Ppu046	Farrell et al. (2012)	6-FAM	2	4 4	41459422 37989476	1.90E−138 2.20E−42	(TG)_10_	332	5	61	F: TCGTCTGATTTGTATTGTTCTT R: TGACACACAGGTTTGGAA	173–182	0.64	0.61	−0.0224
Ppu047	Farrell et al. (2012)	HEX	8	6 6	28674879 27151910	1.60E−103 1.60E−61	(TC)_10_	292	4	57	F: TGCAGCTTTAATTGCAACAGCTAATC R: AGCGCTCAGGTCTGAATGAGTTC	288–294	0.58	0.55	−0.0189
Ppu048	Farrell et al. (2012)	HEX	2	1 1A	4758084 3982690	3.20E−77 2.70E−75	(CT)_10_	373	6	61	F: TGCAGCATTCTTCGCAGCTA R: AACACACTGAGCGTCGTTTTATCA	222–232	0.52	0.47	−0.0552
Ppu049	Farrell et al. (2012)	6-FAM	11	1 1	163634502 65490386	3.00E−08 2.10E−14	(GA)_12_	167	21	61	F: AACTTCAAAGACTTCTGCAAAGTTATTCTTC R: TGAACTTACACTGGTGAACTAACTTTCTCTC	226–411	0.63	0.92	0.1866
Ppu054	Farrell et al. (2012)	6-FAM	3	8 8	15987120 11832348	2.00E−25 7.50E−48	(GT)_5_	375	3	61	F: GCACCGCAGAAGTTGATAAG R: CTGAGGTGCTCATGGTTACAG	283–289	0.01	0.01	−0.0006
Ppu055	Farrell et al. (2012)	6-FAM	2	1 1	196624265 85953917	8.80E−13 7.90E−12	(AGAAAGAA)_7_	62	3	61	F: TGGAGCTTAACATCTACAAATGC R: TTGGCTTTCTCTTATCCATCAC	269–278	0.11	0.35	0.5762*
Ppu056	Farrell et al. (2012)	HEX	5	22 22	690245 1590011	1.00E−22 1.50E−06	(CA)_8_	356	7	59	F: CCTCTGGCAAATACTCAATGC R: CACTGGAAAGGTCAGGAAGC	168–205	0.70	0.62	−0.0675
Ppu057	Farrell et al. (2012)	6-FAM	2	6 6	20079681 18076829	3.70E−21 8.50E−28	(GA)_8_	292	16	61	F: TGCAGTGCAATGTGTGTGACC R: CCTGCTGTGAAATCTACCCATCC	328–381	0.91	0.89	−0.0139
Ppu058	Farrell et al. (2012)	6-FAM	13	Z Z	6052811 37214802	1.30E−27 1.30E−45	(GT)_14_	272	7	63	F: AGTAGCTGCCAATCCACAGG R: TCTCCTGCTTGGCCTCTTT	221–233	0.12	0.81	0.7399*
Ppu059	Farrell et al. (2012)	HEX	6	1 1	121754800 12955004	2.20E−51 2.60E−34	(GT)_8_	156	4	62	F: TCTACTGAGCTCACAGAAACAAAGGAAC R: CTGACTCATGATGCCTCATCTCG	261–264	0.21	0.66	0.5285*
Ruff1	Thuman et al. (2002)	NED	4	– –	– –	– –	(ATCT)_12_	359	7	56	F: TTTCCAAGAGACCAGCAATAAG R: GATTGCTTTGGCTGGAGATG	180–204	0.56	0.61	0.0548
Ruff4	Thuman et al. (2002)	NED	7	– –	– –	– –	(AACT)_3_(AAAT) (AAACT)	224	2	56	F: CAGGAAGTTGTCAATGAAGCTC R: CACGGAGGAACAAGTAAATGAG	238–242	0.15	0.23	0.2220
Ruff5	Thuman et al. (2002)	HEX	9	Z Z	– 71772280	– 6.50E−05	(ATCT)_12_	298	6	56	F: GGTCTGAATATAAGATTTCCTTGG R: AGAATAACCTGGTGCATCTTTC	127–165	0.26	0.62	0.4086*
Ruff6	Thuman et al. (2002)	6-FAM	8	– –	– –	– –	(TGGA)_6_ (TAGA)_14_	279	11	57	F: GAAACCTTCCCATCAACAGAGTA R: CAGAATGAAATATAGTTGCAGCAC	149–186	0.82	0.82	−0.0056
Ruff8	Thuman et al. (2002)	6-FAM	7	Z Z	– –	– –	(CTAT)_10_ (CTACC)	229	11	56	F: ATCTTGCAGGAATCAAAAATGTG R: TGGCTGTCATTTACTCTGTGTTG	92–151	0.41	0.83	0.3337*
Ruff12	Thuman et al. (2002)	6-FAM	8	– –	– –	– –	(AC)_7_ AA(AC)_4_ AA(AC)_3_	255	14	57	F: ATTCCAAACAAATTGCCTAAGG R: CGCTGGAAAAGGTGTTTAGGT	206–263	0.83	0.87	0.0225
Ruff50	Thuman et al. (2002)	HEX	5	18 18	7536672 2604207	1.80E−14 1.60E−16	(GT)_24_	360	4	59	F: GCTGTCAATATGCCATTGGTAACAT R: TTGCAACAGAAACCCATATAAGCAT	138–148	0.52	0.48	−0.0342
Phil2	Verkuil et al. (2012)	6-FAM	2	1 1	114546517 5593870	1.20E−17 4.70E−52	(AG)_28_	262	13	61	F: TGAAGGTTTGTCACTGCAAGA R: GCTTAAAGATTACTTGGGGGAG	208–245	0.59	0.87	0.1960
Phil9	Verkuil et al. (2012)	NED	10	2 2	990418 3872656	9.30E−38 3.50E−37	(AG)_12_	316	6	60	F: GACCACCCAAAGCCCTATAA R: GACCACCCAAAGCCCTATAA	174–184	0.38	0.42	0.0514
Chmo21	St. John et al. (2007)	NED	5	6 6	30023163 28670266	3.30E−92 4.50E−103	(GT)	258	3	59	F: ACTTCATGCAATTAAGTAATCAGAA R: CCTGAAAGTAAGACCTCTCTGG	170–182	0.49	0.54	0.0515
RGB18	Küpper et al. (2008)	NED	3	9 9	15167245 15985365	1.40E−42 1.80E−49	(GT)	375	2	61	F: TGTTCTGAAAGGGCTGCTCATAGTA R: GCATACCTTGCAAGTAGCATCATGT	192–194	0.01	0.01	−0.0004
SnipeB2	Saether et al. (2007)	NED	9	1 1	85466778 94732069	1.10E−48 9.30E−52	(GATA)	114	3	56	F: CTGTACTTGGGCATCTTCCAAGC R: GCAGGATATGGAGGCACTTGAAAT	143–213	0.47	0.43	0.0441
PGT83	Blomqvist et al. (2010)	6-FAM	1	12 12	11733960 12011983	9.40E−31 4.90E−35	(GT)	293	3	58	F: AATCCGTTTCTGGGGACTGGG R: TGCCTAATGCTGACTCACACC	149–152	0.33	0.32	−0.0394
TG22-001	Dawson et al. (2010)	HEX	6	22 22	529247 1428098	1.10E−118 3.00E−153	(AT)	37	3	62	F: TTGGATTTCAGAACATGTAGC R: TCTGATGCAAGCAA	246–252	0.27	0.61	0.3820*
TG05-053	Dawson et al. (2010)	6-FAM	9	5 5	59348193 61276203	7.60E−120 2.30E−161	(AT)	215	2	60	F: GCATCATCTGGTTGAACTCTC R: ACCCTGTTTACAGTGAGGTGTT	210–212	0.18	0.22	0.0982

Summary of genotyping results and predicted genome locations of 58 ruff microsatellite loci. Of the 58 polymorphic loci characterized, 55 could be assigned a location in the chicken genome and 53 in the zebra finch genome. MP, the PCR multiplex set used in genotyping; *n*, number of individuals amplified and genotyped; *A*, number of alleles observed; *H*_O_, observed heterozygosity, *H*_E_, expected heterozygosity (calculated from *n*, using CERVUS v3.0); *markers with null alleles. Null allele frequencies were calculated using the original genotypes and are based on the excess of homozygous individuals. Excesses of homozygotes are probably due to nonrandom population structure caused by captive breeding that included matings between full sibs and second-order relatives (half-sibs and closer relatives).

1 The location of each microsatellite sequence was assigned in the chicken (*Gallus gallus*; v 2.1, May 2006 ENSEMBL release) and zebra finch (*Taeniopygia guttata*; December 2011 ENSEMBL Release 65) genomes based on sequence similarity (see Dawson et al. 2006, 2007).

### DNA extraction and genotyping

We obtained DNA from blood and frozen tissue that had been collected from individuals and stored in absolute ethanol. Genomic DNA was extracted using an ammonium acetate precipitation method (Nicholls et al. 2000; Richardson et al. 2001). Each 2-μL PCR contained approximately 10 ng genomic DNA, 0.2 μmol/L of each primer, and 1 μL Qiagen Multiplex PCR Mix (Qiagen Inc). PCR amplification was performed using a DNA Engine Tetrad 2 Thermal Cycler (MJ Research, BioRad, UK) with the profile: 15 min at 95°C, followed by 35 cycles of 94°C for 30 sec, annealing temperature (Table 1) for 90 sec and 72°C for 1 min, then a final step of 60°C for 10 min. PCR products were loaded onto an ABI3730 Genetic Analyzer (Applied Biosystems) using ROX500 size standards, and genotypes were scored with GENEMAPPER v4.0 software (Applied Biosystems). Observed and expected heterozygosities were calculated using CERVUS v3.0 (Kalinowski et al. 2007; Table 1). Deviations from Hardy–Weinberg equilibrium and linkage disequilibrium were assessed using GENEPOP v.4.0 (Rousset 2008). Four loci identified in ruffs (*Ppu042*, *Ppu023*, *Ppu033*, and *Ppu012*; Farrell et al. 2012), and one primer set from another species (*Chmo06*; St. John et al. 2007) failed to amplify in the genotyping multiplexes and were excluded from further analysis.

### Pedigree assembly and linkage mapping

Parentage assignment was performed using genotypic data for all 58 microsatellite markers in 381 individuals (including 8% data replicates) using CERVUS v.3.0. The resulting parentage assignments were compared with the previous pedigree, held by DBL and SBM, for inconsistencies. Grandparent–Parent–Offspring genotypic inconsistences arising from incorrect parentage assignment or microsatellite genotyping errors were detected through a three-generation Pedigree Program (K. W. Kim, unpublished) and either resolved by rechecking the parentage and past genotyping records held by DBL and SBM, reviewing raw allele peaks on GENEMAPPER v.4.0 or, in any remaining cases of uncertainty, rescored as untyped.

Linkage analysis was performed using a version of CRIMAP v.2.4 (Green et al. 1990), modified by Xuelu Liu (Monsanto) to accommodate large numbers of markers in complicated pedigrees. Prior to input into CRIMAP, CRIGEN was used to simplify the pedigree and omit any noninformative individuals. A two-point linkage analysis of all markers was then performed based on a LOD score > 3.0. Markers were also assumed to be linked if they were supported by a LOD > 2.0 and an expectation of linkage based on *a priori* knowledge (Slate et al. 2002), that is, linkage was expected based on BLAST search (Altschul et al. 1997) and assignment of chromosomal location in chicken and zebra finch (Dawson et al. 2006, 2007). Linkage groups were created using AUTOGROUP and markers belonging to the same linkage group were analyzed using the BUILD command. PUK_LIKE_TOL and PK_LIKE_TOL values were lowered from 3.0 to 2.0, and then 2.0 to 1.0, and the BUILD command rerun until no further markers were added. Marker order was determined and confirmed by the FLIPS command, where new marker orders were tested against alternative orders to determine whether they fitted the data. Recombination frequencies and positions of all loci in linkage groups were visualized using the CHROMPIC function. During map construction, both sex-averaged and sex-specific maps were built; however, only the sex-averaged maps per linkage group are presented, with map distances based on the Kosambi mapping function.

### Genome coverage

The mean marker spacing was calculated by dividing the total length of the map by the number of intervals. Average intramarker spacing for each linkage group was calculated by dividing the length of each linkage group by the total number of intervals on that linkage group. Linkage map coverage was calculated by summing the difference in base-pair position in chicken of the first and last interval on each linkage group, and dividing by the total base-pair length of the chicken genome (∼1.07 Gb; Ensembl database http://www.ensembl.org/Gallus_gallus/index.html).

## Results and Discussion

Based on comparative mapping methods of microsatellite loci homologous to the ruff, chicken, and zebra finch, homologs of 55 of the 58 typed microsatellite loci were assigned predicted chromosomal locations in the chicken genome and 53 were assigned locations in the zebra finch (Table 1). Five ruff microsatellite sequences (*Ppu008*, *Ruff1*, *Ruff4*, *Ruff6*, and *Ruff12*) could not be assigned predicted chromosomal locations in either genome based on sequence similarity.

**Figure 1:**
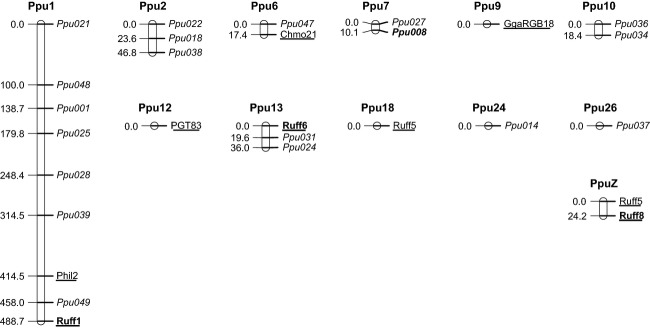
A first-generation linkage map of the ruff (*Philomachus pugnax*) consisting of seven linkage groups and five single markers ordered by homologous chromosome size. Positions given in centimorgan. Linkage groups with marker order supported by either LOD > 3.0, or LOD > 2.0 in agreement with a predicted location are presented, as well as single-marker loci assigned locations on chromosomes. Loci in italics are described in Farrell et al. (2012); loci underlined are cross-utility shorebird loci (Thuman et al. 2002; Küpper et al. 2008; St. John et al. 2007; Blomqvist et al. 2010; Verkuil et al. 2012). Loci in bold are four loci previously unassigned a chromosomal location by a predictive mapping method that are here assigned a chromosomal location via linkage analysis.

The first-generation linkage map of the ruff consisted of 23 microsatellite markers resolved into 7 linkage groups (Ppu1, Ppu2, Ppu6, Ppu7, Ppu10, Ppu13, and PpuZ) homologous to chicken and zebra finch chromosomes. Each linkage group was numbered according to the homologous chicken and zebra finch chromosome number (with the prefix Ppu; Fig. 1). An additional five loci were not expected to be linked to any other marker, based on predicted genomic locations. This expectation was confirmed by the two-point analysis, and so these were treated as linkage groups with a single marker (Fig. 1). The remaining 30 markers were expected to form linkage groups, but were found to be unlinked to all other markers. The map covers 641.6 cM with an average spacing of 22.9 cM. The size of linkage groups, ignoring those that consisted of a single marker, ranged from 10.1 to 488.7 cM. The number of markers per linkage group varied from 2 to 9. The intermarker interval for each linkage group varied from 5.0 to 54.3 cM, with a mean of 16.7 cM.

Four of the markers that lacked predicted genomic locations were subsequently assigned to chromosomes on the basis of the linkage mapping: *Ruff1*, *Ruff6*, *Ppu008,* and *Ruff8* were assigned to chromosomes Ppu1, Ppu13, Ppu7, and Z, respectively. *Ruff8* was known to be Z-linked from previous work by Thuman et al. (2002); however, its genomic location on chromosome Z is reported here for the first time. Chromosomes Ppu1A, Ppu3, Ppu4, Ppu5, Ppu8, Ppu11 and Ppu22 were all predicted to contain more than one typed marker; yet, linkage groups could not be formed. There are two possible explanations for the failure to assign the markers to these chromosomes. First, the pedigree may have been insufficiently powerful to map all linked markers, especially if they were relatively far apart on a chromosome. Second, the predicted chromosomal locations may not be an accurate indication of the true locations; in other words, synteny may not be highly conserved between ruffs and other birds. Given that no mapped markers were assigned to locations other than their predicted locations, we believe that the failure to assign markers to these chromosomes is an issue of power rather than poorly conserved synteny.

Following the methods of Backström et al. (2008), we used available physical data on the chicken genome to calculate the proportion of the ruff genome covered by the map. The distance on the chicken genome assembly between the homologs of the most distal markers on each ruff linkage group was estimated, and summing across chromosomes was found to be 270 Mb, or 26% of the total ∼1.07 Gb chicken genome (Ensembl database http://www.ensembl.org/Gallus_gallus/index.html). However, additional sequence is covered by the ruff map if the five chromosomes with single markers and the sequence immediately beyond the first and last markers on each linkage group are included. Assuming the ruff has a similar genome size to the chicken (http://www.genomesize.com/), it may be estimated that our map covers 30–35% of the ruff genome. The proportion of the total genetic (i.e., recombination) length of the ruff genome covered by the map is harder to assess, as the microchromosomes are mostly unmapped. Although microchromosomes are physically short and contribute little to the physical genome size, they each have an obligate crossing-over event during meiosis, which contributes 50 cM to the total map length (Jones and Frankin 2006). Thus, compared with its coverage of the physical genome, the map must cover a lower proportion of the total linkage (recombination) map length of the ruff genome.

**Figure 2:**
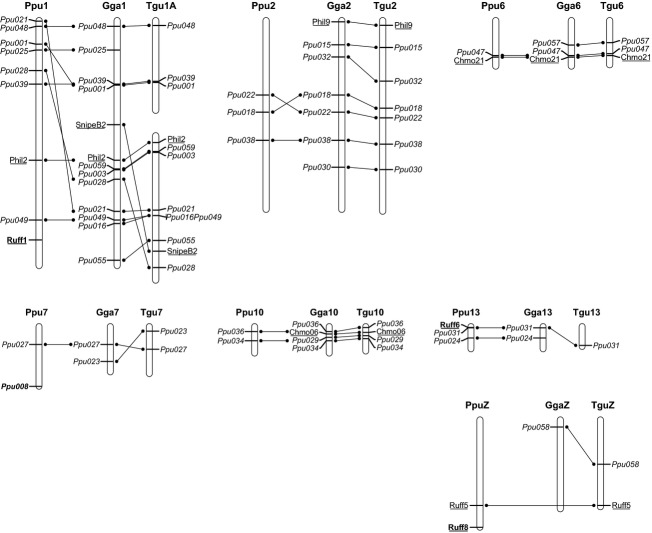
A comparative map of microsatellite loci in ruff (Ppu; *Philomachus pugnax*), chicken (Gga; *Gallus gallus*), and zebra finch (Tgu; *Taeniopygia guttata*) homologous chromosomes. Distinctions between loci in italics, bold font, and underlined are explained in the legend of Figure 1. Three possible intrachromosomal rearrangements between the homologs of chicken, zebra finch and ruff microsatellite loci are reported here for the first time (chr1: loci *Ppu001*, *Ppu021,* and *Ppu028*; chr2: loci *Ppu018* and *Ppu022*; chr7: loci *Ppu023* and *Ppu027*).

Despite the highly conserved synteny generally believed to exist among avian genomes (Griffin et al. 2007), comparative mapping among the homologs of chicken, zebra finch, and ruff microsatellite loci results in three possible intrachromosomal rearrangements being reported for the first time on chromosome 1 (involving loci *Ppu001*, *Ppu021,* and *Ppu028*), chromosome 2 (loci *Ppu018* and *Ppu022*) and chromosome 7 (loci *Ppu023* and *Ppu027*; Fig. 2). These types of rearrangements were once thought to be relatively rare in birds (Stapley et al. 2008). However, with the recent sequencing of the turkey (*Meleagris gallopavo*) genome, comparative analyses between the turkey, zebra finch (*Taeniopygia guttata*), and chicken (*Gallus gallus*) have identified a large number of intrachromosomal rearrangements, reflective of avian genome evolution (Skinner and Griffin 2012). Therefore, these regions are of evolutionary interest in the ruff.

In summary, the map of seven linkage groups and length 641.6 cM covers an estimated 30–35% coverage of the ruff genome. It is the first linkage map of any shorebird species and will be of utility, even at this low density, as previous studies with approximately 30% map coverage have met with some success in the mapping of phenotypic loci (Miwa et al. 2006). Thus, this map has the potential to provide an essential framework for further studies mapping important behavioral and plumage traits in this species.

## References

[b1] Altschul SF, Madden TL, Schäffer AA, Zhang J, Miller W, Lipman DJ (1997). Gapped BLAST and PSI-BLAST: a new generation of protein database search programs. Nucleic Acids Res.

[b2] Backström N, Karaiskou N, Leder EH, Gustafsson L, Primmer CR, Qvarnström A (2008). A gene-based genetic linkage map of the collared flycatcher (*Ficedula albicollis*) reveals extensive synteny and gene-order conservation during 100 million years of avian evolution. Genetics.

[b3] Blomqvist D, Pauliny A, Larsson M, Flodin LÅ (2010). Trapped in the extinction vortex? Strong genetic effects in a declining vertebrate population. BMC Evol. Biol.

[b4] Dale J, Lank DB, Reeve HK (2001). Signalling individual identity versus quality: a model and case studies with ruffs, queleas, and house finches. Am. Nat.

[b5] Dawson DA, Burke T, Hansson B, Pandhal J, Hale MC, Hinten GH (2006). A predicted microsatellite map of the passerine genome based on chicken–passerine sequence similarity. Mol. Ecol.

[b6] Dawson DA, Åkesson M, Burke T, Pemberton JM, Slate J, Hansson B (2007). Gene order and recombination rate in homologous chromosome regions of the chicken and a passerine bird. Mol. Biol. Evol.

[b7] Dawson DA, Horsburgh GJ, Küpper C, Stewart IRK, Ball AD, Durrant KL (2010). New methods to identify conserved microsatellite loci and develop primer sets of high utility – as demonstrated for birds. Mol. Ecol. Resour.

[b8] Farrell LL, Dawson DA, Horsburgh GJ, Burke T, Lank DL (2012). Isolation, characterisation and predicted genome locations of ruff (*Philomachus pugnax*, AVES) microsatellite loci. Conserv. Genet. Resour.

[b9] Green P, Falls K, Crooks S (1990). CRIMAP Documentation, version 2.4.

[b10] Griffin DK, Robertson LBW, Tempest HG, Skinner BM (2007). The evolution of the avian genome as revealed by comparative molecular cytogenetics. Cytogenet. Genome Res.

[b11] Hogan-Warburg AJ (1966). Social behavior of the ruff (*Philomachus pugnax*. Ardea.

[b12] Höglund J, Lundberg A (1989). Plumage color correlates with body size in the ruff (*Philomachus pugnax*. Auk.

[b13] Holleley CE, Geerts PG (2009). Multiplex Manager 1.0: a cross-platform computer program that plans and optimizes multiplex PCR. Biotechniques.

[b14] Jones GH, Frankin C (2006). Meiotic crossing-over: obligation and interference. Cell.

[b15] Jukema J, Piersma T (2006). Permanent female mimics in a lekking shorebird. Biol. Lett.

[b16] Kalinowski ST, Taper ML, Marshall TC (2007). Revising how the computer program CERVUS accommodates genotyping error increases success in paternity assignment. Mol. Ecol.

[b17] Küpper C, Burke T, Székely T, Dawson DA (2008). Enhanced cross-species utility of conserved microsatellite markers in shorebirds. BMC Genomics.

[b19] Lank DB, Smith CM, Hanotte O, Burke T, Cooke F (1995). Genetic polymorphism for alternative mating behaviour in lekking male ruff, *Philomachus pugnax*. Nature.

[b20] Lank DB, Farrell LL, Burke T, Piersma T, McRae SB (2013). A dominant allele controls development into female mimic male and diminutive female ruffs. Biol. Lett.

[b21] Miwa M, Inoue-Murayama M, Kobayashi N, Kayang BB, Mizutani M, Takahashi H (2006). Mapping of panda plumage colour locus on the microsatellite linkage map of the Japanese quail. BMC Genet.

[b22] Nicholls JA, Double MC, Rowell DM, Magrath D (2000). The evolution of cooperative and pair breeding in thornbills *Acanthiza* (Pardalotidae). J. Avian Biol.

[b18] Richardson DS, Jury FL, Blaakmeer K, Komdeur J, Burke T (2001). Parentage assignment and extra-group paternity in a cooperative breeder: the Seychelles warbler (*Acrocephalus sechellensis*. Mol. Ecol.

[b23] Rousset F (2008). GENEPOP'007: a complete re-implementation of the GENEPOP software for Windows and Linux. Mol. Ecol. Resour.

[b24] Saether SA, Fiske P, Kålås JA, Kuresoo A, Luigujoe L, Piertney SB (2007). Inferring local adaptation from Q_ST_–F_ST_ comparisons: neutral genetic and quantitative trait variation in European populations of great snipe. J. Evol. Biol.

[b25] Skinner BM, Griffin DK (2012). Intrachromosomal rearrangements in avian genome evolution: evidence for regions prone to breakpoints. Heredity.

[b26] Slate J, Visscher PM, MacGregor S, Stevens DR, Tate ML, Pemberton JM (2002). A genome scan for QTL in a wild population of red deer (*Cervus elaphus*. Genetics.

[b27] St. John J, Kysela RF, Oyler-McCance SJ (2007). Characterization of microsatellite loci isolated in mountain plover (*Charadrius montanus*. Mol. Ecol. Resour.

[b28] Stapley J, Birkhead TR, Burke T, Slate J (2008). A linkage map of the zebra finch *Taeniopygia guttata* provides new insight into avian genome evolution. Genetics.

[b29] Thuman KA, Widemo F, Piertney SB (2002). Characterization of polymorphic microsatellite DNA markers in the ruff (*Philomachus pugnax*. Mol. Ecol. Notes.

[b30] Van Rhijn JG (1973). Behavioural dimorphism in male ruffs *Philomachus pugnax* (L.). Behaviour.

[b31] Verkuil YI, Piersma T, Jukema J, Hooijmeijer JC, Zwarts EW, Baker AJ (2012). The interplay between habitat availability and population differentiation: a case study on genetic and morphological structure in an inland wader (Charadriiformes). Biol. J. Linn. Soc.

[b32] Voorrips RE (2002). MapChart: software for the graphical presentation of linkage maps and QTLs. J. Hered.

